# Social Anxiety among Middle-Aged Teachers in Secondary Education Schools

**DOI:** 10.3390/ejihpe14080158

**Published:** 2024-08-18

**Authors:** Lihan Guo, Ratanaporn Awiphan, Tinakon Wongpakaran, Penkarn Kanjanarat, Danny Wedding

**Affiliations:** 1 Master of Science Program (Mental Health), Multidisciplinary and Interdisciplinary School (MIdS), Chiang Mai University, Chiang Mai 50200, Thailand; jasmineguo222@gmail.com (L.G.); danny.wedding@gmail.com (D.W.); 2 Department of Pharmaceutical Care, Faculty of Pharmacy, Chiang Mai University, Chiang Mai 50200, Thailand; 3 Pharmacoepidemiology and Statistics Research Center (PESRC), Faculty of Pharmacy, Chiang Mai University, Chiang Mai 50200, Thailand; 4 Department of Psychiatry, Faculty of Medicine, Chiang Mai University, Chiang Mai 50200, Thailand; 5 Department of Clinical and Humanistic Psychology, Saybrook University, Pasadena, CA 91103, USA; 6 Department of Psychology, University of Missouri-Saint Louis, St. Louis, MO 63121, USA

**Keywords:** social anxiety symptoms, middle-aged teachers, secondary education, prevalence, Thailand

## Abstract

Background: This study aimed to investigate the prevalence of social anxiety symptoms (SASs) and its associated factors among middle-aged teachers in secondary education schools. Methods: A cross-sectional survey was conducted from December 2023 to March 2024 among 341 secondary education schoolteachers aged 45–59 in Chiang Mai, Thailand, involving an online survey. Effects of psychosocial variables on SASs were investigated, including attachment anxiety, attachment avoidance, neuroticism and extraversion personality traits, loneliness, perceived social stress, job burnout, and anxiety and depression. Multiple linear regression was used to identify predictors of SASs. Results: This study found that 98 out of 341 (28.7%) teachers presented SASs. Multiple linear regression analysis showed that marital status (β = 0.103, 95% CI [0.437, 3.404]), income (β = 0.087, 95% CI [0.049, 3.758]), extraversion (β = −0.179, 95% CI [−0.573, −0.198]), attachment anxiety (β = 0.165, 95% CI [0.106, 0.359]), attachment avoidance (β = 0.145, 95% CI [0.066, 0.243]), depression (β = 0.242, 95% CI [0.248, 0.862]), loneliness (β = 0.182, 95% CI [0.099, 0.580]), and perceived social stress (β = 0.235, 95% CI [0.131, 0.373]) were significant predictors of SASs, explaining 51.1% of the variance. Conclusion: This study discovered a relatively high prevalence of SASs among middle-aged secondary schoolteachers.

## 1. Introduction

Teachers significantly influence individual and societal development, underscoring the importance of their mental health. A scoping review found that among teachers, burnout prevalence ranged from 25.12% to 74%, stress from 8.3% to 87.1%, anxiety from 38% to 41.2%, and depression from 4% to 77% [1], particularly during the COVID-19 pandemic [2]. Previous studies showed that work-related factors significantly affect teachers’ mental health, including excessive workload, long working hours, large classroom sizes, poor working conditions, role conflict, and lack of resources [3,4,5,6]. Social anxiety symptoms (SASs) were reported as one of the significant mental health issues among schoolteachers [7,8]. A study of SASs in Chinese teachers showed that female teachers had significantly higher social anxiety scores than male teachers, while teachers in their 30s and 40s were more likely to experience social anxiety because they were on the rise in their careers and work took up most of their social time. In addition, teachers with lower incomes were more likely to experience SASs [7]. SASs are described as feelings of intense anxiety, fear of being judged or negatively evaluated by others, or rejected in a social or performance situation. Some examples of SASs include physical symptoms such as shaking, blushing, and sweating, avoidance of social situations, fear of public speaking, eating, or drinking. SASs may be overlooked because it is presented on a continuous spectrum from the absence of social fear through normal anxiety and shyness to social anxiety disorder (SAD) [9].

Due to the nature of their profession, teachers may be prone to social anxiety from frequent public speaking and interactions with students, colleagues, and parents. Although limited studies exist on the predictors of social anxiety disorder among teachers [7,8,10], work-related issues have been identified as a contributing factor [1]. Those work-related factors potentially contributing to social anxiety in teachers include the constant pressure to meet educational standards and evaluations by peers, administrators, and parents, which can increase anxiety [11]. Managing diverse classroom behaviors and maintaining discipline can be stressful and anxiety-inducing [7,8]. Dealing with parents, especially in conflict situations, can be challenging and trigger social anxiety. Teachers may feel immense pressure to perform well and ensure their students succeed, contributing to anxiety. The extensive workload, including lesson planning, grading, and extracurricular activities, can lead to burnout and mental health issues, including anxiety. A study showed that job burnout was associated with social anxiety among primary schoolteachers in Greece [8]. Particularly in middle-aged teachers, they often grapple with a combination of career pressures, family responsibilities, and the physical and emotional aspects of aging. They tend to perceive stress [12], and job burnout related to workload [8,12]. A study of primary and secondary schoolteachers in China [7] indicated that teleworking was more likely to cause social anxiety among teachers than non-teleworking, while work intensity promoted SASs and online social support could reduce the probability of SASs.

In addition to workload and job burnout, other predisposing factors, underlying traits, or conditions that make an individual more susceptible to developing social anxiety symptoms include insecure attachment, which can be categorized as attachment anxiety and attachment avoidance, with attachment anxiety being an individual’s attempt to try to shorten their distance from others and seek support due to a fear of rejection or disapproval by others [13]. On the other hand, attachment avoidance reflects the extent to which individuals perceive others as untrustworthy and avoid relying on them [14]. Current evidence confirmed that insecure attachment, that is, attachment anxiety and attachment avoidance, was strongly associated with symptom severity of social anxiety [15,16,17].

Personality traits based on the five-factor model such as neuroticism is a predisposing temperament dimension marked by stress reactivity that leads to frequent negative emotional experiences and implies a person’s incapacity to manage as well as cope with beliefs about challenging events [18], and extraversion, manifested in outgoing, talkative, energetic behaviors. Introversion, in contrast, is associated with reflective and reserved behavior [19], and it was found to have a significant relationship with social anxiety. Neuroticism had a positive relationship with social anxiety, whereas extraversion had a negative relationship [20,21].

In addition to the predisposing factors, contributing factors such as loneliness, which exists when there is a discrepancy between the relationships one wishes to have and the relationships one currently has [22], are important variables. There is a reciprocal relationship between loneliness and social anxiety over time [23,24]. Other precipitating factors, such as generalized anxiety and depression, can often present with comorbidities [25]. The severity of depression was correlated with the severity of social anxiety [25,26].

Based on current knowledge, most studies on social anxiety focused on students rather than teachers, and prevalence and associated factors in students could not be used to describe the teacher population. The notable gap in research is twofold. Firstly, based on the very limited results of social anxiety symptoms among middle schoolteachers, it is evident that this mental health problem is overlooked; therefore, it is worth exploring and increasing our knowledge on this important population [7]. As we know the characteristics of teachers’ work, teachers often carry high social expectations and the pressure of work and family; if the mental health status of teachers was neglected, it would have a negative impact on teaching and learning outcomes, specifically on the teacher–student relationship, students’ physical and mental health, and educational achievement, so teachers’ mental health needs to be paid attention to in a timely manner [27]. Secondly, in addition to learning about the prevalence of social anxiety symptoms, it is important to examine further factors associated with such mental health problems, including personality traits, work-related factors, attachment styles, loneliness, perceived social stress, anxiety, and depression, as the independent variables. Based on the above research, the study aimed to investigate the prevalence of social anxiety symptoms and their associated factors in middle-aged secondary education teachers in Chiang Mai, Thailand. We hypothesized that the prevalence of social anxiety symptoms might not be different from other populations considering other involved factors. We also hypothesized that social anxiety symptoms were positively associated with high levels of job burnout, neuroticism, extraversion, attachment anxiety, attachment avoidance, loneliness, perceived social stress, anxiety, depression, and teaching evaluation.

## 2. Materials and Methods

### 2.1. Study Design

We implemented a cross-sectional study to explore the prevalence of social anxiety symptoms and associated factors among secondary education teachers in Chiang Mai, Thailand from December 2023 to March 2024. The study investigated the prevalence of social anxiety symptoms and associated factors among secondary education teachers in public, private, and demonstration schools in Chiang Mai, Thailand. Based on a biopsychosocial model [28], this study investigated biological, psychological, and social determinants of social anxiety symptoms. The BPS model provides a comprehensive approach to the understanding of the condition and underlying causes of social anxiety symptoms.

### 2.2. Participants

We collected data involving secondary education schoolteachers from 34 public schools, 28 private schools, and 1 demonstration school in Chiang Mai, Thailand, from 8 December, 2023, to 27 March, 2024. Inclusion criteria included secondary education teachers in Chiang Mai, Thailand, aged 45–59 years, who had worked in a teaching-related job, could read Thai fluently, and provided online consent to participate in the study. There was no restriction on the sex of the participants. Exclusion criteria were participants younger than 45 or older than 59 years, missing all items in at least one instrument, and with duplicate data entry. We recruited participants in the following ways: first, we cooperated with the Chiang Mai Secondary Education Service Area Office, which sent invitations for the study to the schools’ management staff. The purpose and process of the study were explained in detail through paper documents, and after the school administration agreed, teachers were invited to join the study voluntarily. For teacher demonstration schools and private schools, the researcher explained the purpose and process of the study in detail over the phone and via email to the school administration; once agreed to cooperate, the invitation flyer, QR codes and the links to the questionnaire were distributed to the teachers by e-mail and Line to invite teachers to participate in the study voluntarily. Study invitations were also posted through education forums and teachers’ social net-working platforms to attract interested teachers to participate. All participants were re-quired to read and agree to the informed consent form before completing the online questionnaire. The average age of the teachers was 50.22 years (SD = 4.50) and about half of the respondents (*n* = 175, 51.3%) were between the ages of 45 and 49. Of all respondents, 235 were females (68.9%), and 216 respondents (63.3%) were educated with a bachelor’s degree or below. More than half of the participants (61.6%) were in a relationship. Most respondents (76.2%) reported earning more than 20,000 baht or more monthly, 546 USD. Among all, 83.9% of respondents reported that their health status on the day they completed the questionnaire was in the range of 51–100.

### 2.3. Procedure

**Figure 1:**
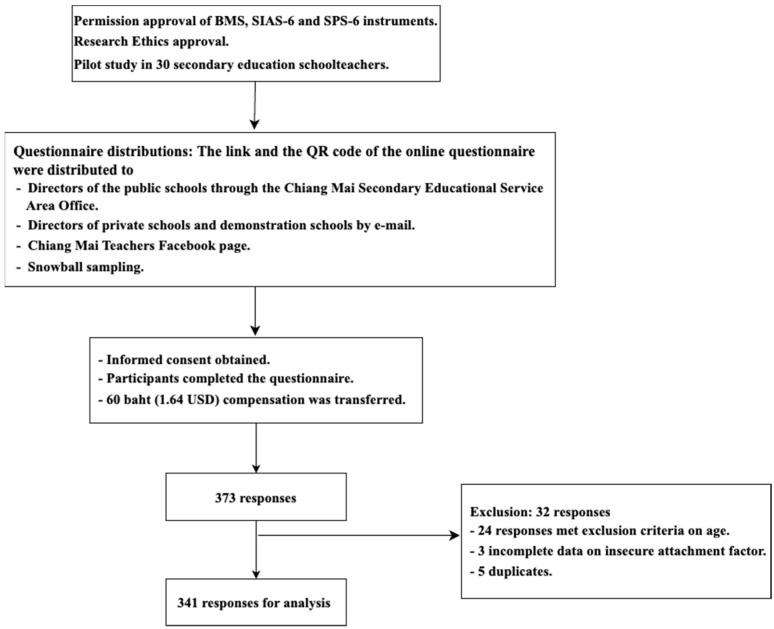
Flow chart of the study.

This study was approved by the Ethics Committee (EC), Faculty of Pharmacy, Chiang Mai University, Thailand, in July 2023, and the revision was approved in October 2023. This study was approved by the Ethics Committee (EC) of the Faculty of Pharmacy, Chiang Mai University, Thailand, in July 2023, and the revision was approved in October 2023. The link to the online questionnaire and the QR code were distributed to the directors of public schools through the Chiang Mai Secondary Education Service Area Office, and the online questionnaire was forwarded to teachers accordingly if the request was approved by the school director. For private schools and demonstration schools, the researchers sent the link and QR code of the online questionnaire to the school directors via email. In addition, the researchers posted the link to the online questionnaire in the Chiang Mai Teachers group on Facebook and used a snowballing approach to recruit more participants. The first page of the online questionnaire was the “Participant Information Sheet (PIS)” and the “Informed Consent Form (ICF)”, and the participants’ personal information would not be used for any other purpose. In addition, all questionnaires’ data were kept confidential according to the ethical guidelines to protect the participants’ identities. All participants who volunteered to participate in this study completed the questionnaire and provided a bank account or PromptPay number were compensated with 60 baht (1.64 USD) (Figure 1).

### 2.4. Measurements

#### 2.4.1. Social Interaction Anxiety Scale and Social Phobia Scale (SIAS-6 and SPS-6)

The Social Interaction Anxiety Scale and the Social Phobia Scale (SIAS and SPS), developed by Mattick and Clarke (1998) [29], are self-report tools designed to measure two related but different aspects of social anxiety. The shorter SIAS and SPS (SIAS-6 and SPS-6) were developed by Lorna Peters and colleagues [30]; they include items that are rated on a 5-point Likert scale, with 0 representing not at all characteristic or true of me and 4 representing extremely characteristic or true of me. Scores are calculated by summing the 6 ratings for each scale, and the higher the total scores, the higher the social anxiety. The ideal cutoff score for social anxiety was 2 or higher on the SPS-6 and 7 or higher on the SIAS-6. The scale has been validated in many countries with good reliability, and SIAS-6 and SPS-6 were translated into the Thai language and approved by developers from Macquarie University. Internal reliability of the instrument was tested with Thai secondary education teachers with a Cronbach’s alpha of 0.889 for the SIAS subscale and 0.951 for the SPS subscale.

#### 2.4.2. Experience in Close Relationships (ECR-R-10)

The Experiences in Close Relationships (ECR) is a self-report measurement used to assess attachment in adults [31]. This scale modified the original 36 and 18 items of ECR-R by Fraley [32]. The ECR-R-10 includes two subscales: attachment anxiety and attachment avoidance. The Thai ECR-R includes 10 items [33]. Every item was rated on a 7-point Likert scale, ranging from 1 (strongly disagree) to 7 (strongly agree), with 4 being neutral. Each subscale score varied from 5 to 35: the higher the score, the greater the corresponding insecure attachment style. Internal reliability of the instrument was tested with in Thai secondary education teachers with a Cronbach’s alpha of 0.884 for the anxiety subscale and 0.941 for the avoidance subscale.

#### 2.4.3. Zuckerman, Kuhlman, and Aluya Personality Questionnaire (ZKA-PQ)

The ZKA-PQ is a self-report scale developed by Zuckerman, Kuhlman, and Aluya [34,35,36,37]. It has 5 dimensions: aggression, sensation seeking, activity, extraversion, and neuroticism. The short version of ZKA-PQ, comprising 40 items and 8 items for each subscale, was developed using a Thai sample due to the validity problem of ZKA-PQ among the Thai population [38]. Every item is on a 4-point Likert scale, with 1 representing strongly disagree and 4 representing strongly agree. The scores range from 8 to 32 for subscales. A higher score indicates a higher level of the respective trait. Internal reliability of the instrument was tested with Thai secondary education teachers, with Cronbach’s alpha of extraversion, and neuroticism subscales being 0.753 and 0.853, respectively. 

#### 2.4.4. Revised UCLA Loneliness Scale (RULS-6)

The RULS is a 6-item self-rating questionnaire evaluating loneliness, derived from the Revised UCLA Loneliness Scale [39]. Every question uses a 4-point Likert scale, with 1 indicating never and 4 indicating always. The greater the score, the more loneliness is indicated, involving scores from 6 to 24. The instrument’s internal reliability was tested with Thai secondary education teachers, with Cronbach’s alpha of 0.917.

#### 2.4.5. Perceived Stress Scale-10 (PSS-10)

The PSS is a self-assessment scale designed to measure the degree to which life events have been perceived as stressful in the past month [40]. It consists of 10 items, rated on a 5-point Likert scale from 0 (never) to 4 (very often). The overall score varies between 0 and 40. The higher the scores, the greater the perceived stress. The Thai version demonstrates good reliability and validity [41]. The internal reliability of the instrument was tested with in Thai secondary education teachers with Cronbach’s alpha of 0.872.

#### 2.4.6. Outcome Inventory (OI-21)

The OI-21 is a 21-item self-report questionnaire that measures the level of anxiety, depression, somatization, and interpersonal difficulty. Each question was on a five-point Likert scale, with 0 representing never and 4 representing almost always. The total scores ranged from 0 to 55, showing that the higher the score, the higher the symptoms. The OI-21 demonstrates good reliability and validity [42]. Our study only used the anxiety and depression subscales, and the internal reliability of the anxiety and depression subscales was tested with in Thai secondary education teachers with Cronbach’s alpha of 0.831 and 0.910, respectively.

#### 2.4.7. Burnout Measure, Short Version (BMS-10)

The short version of BMS is a self-report tool to measure job burnout, including an individual’s physical, emotional, and mental exhaustion [43,44], with Cronbach’s alpha varying from 0.85 to 0.92. Each question was evaluated on 7-point frequency scales, varying from 1 (never) to 7 (always). The Thai version of BMS-10 was translated and approved by the developers at Ben-Gurion University. In the current study, BMS exhibited the Cronbach’s alpha of 0.921 among Thai secondary education teachers.

#### 2.4.8. Covariates

The present study also included age (45–49, 50–54, and 55–59 years old), sex (male/female), income (≤20,000 (546 USD) or >20,000 Thai Baht per month), education (lower secondary school, high school, associate degree/vocational certificate/or equivalent, bachelor’s degree, master’s degree, and doctoral degree) and marital status (married and living together, not married but living together, and married but not living together. The single category included single, divorced, widowed, separated, and break up).

### 2.5. Statistical Analysis

All statistical analyses were performed using SPSS Statistics (version 26), and a *p*-value < 0.05 was considered significant. Missing variables were handled using multiple imputation. Descriptive analysis described demographic data, mental health outcomes, and SIAS-6 and SPS-6 scores as means and standard deviations. Numbers and percentages were used to summarize sociodemographic variables and the prevalence of social anxiety symptoms. For continuous variables, inferential statistics, *t*-test and ANOVA, were used. The Chi-square test was used to examine differences between demographic variables. The Pearson correlation test was used to determine correlations between continuous variables. Finally, multiple linear regression analysis was used to identify associations of the factors and the study outcome. Testing of the assumptions of linear regression was performed to ensure the validity and reliability of the model. The tests included Linearity (scatter plot), Independence (Durbin–Watson Test), Homoscedasticity (Breusch–Pagan Test), Normality of Residuals (QQ-Plot), and Multicollinearity (Variance Inflation Factor (VIF). R^2^ was calculated to determine the variance explained by the model. 

## 3. Results

### 3.1. Psychological Measures of Participants

The prevalence of social anxiety symptoms in this population was 28.7%. The means and standard deviations of the scores of attachment anxiety, attachment avoidance, neuroticism, extraversion, anxiety, depression, loneliness, perceived social stress, job burnout, and teaching evaluation scores are shown in Table 1.

### 3.2. Social Anxiety Symptoms of Participants by Sociodemographic Characteristics

As shown in Table 2, we found only the education group that had significant differences in social anxiety symptoms.

### 3.3. Pearson Correlations among Mental Health Variables

Pearson’s correlation analysis in Table 3 showed that SIAS and SPS total scores were correlated with attachment anxiety (r = 0.356, *p* < 0.01), attachment avoidance (r = 0.192, *p* < 0.01), neuroticism (r = 0.446, *p* < 0.01), extraversion (r = −0.403, *p* < 0.01), anxiety (r = 0.523, *p* < 0.01) and depression (r = 0.584, *p* < 0.01), loneliness (r = 0.572, *p* < 0.01), perceived social stress (r = 0.463, *p* < 0.01), and job burnout (r = 0.464, *p* < 0.01).

### 3.4. Multiple Linear Regression Results

The results in Table 4 showed that marital status (β = 0.103, *p* = 0.011), income (β = 0.087, *p* = 0.044), attachment anxiety (β = 0.165, *p* < 0.001), attachment avoidance (β = 0.145, *p* = 0.001), extraversion (β = −0.179, *p* < 0.001), depression (β =  0.242, *p* < 0.001), loneliness (β = 0.182, *p* = 0.006), and perceived social stress (β = 0.235, *p* < 0.001) were significant predictors of social anxiety symptoms. Adjusted R^2^ was 0.511, F (14, 309) = 25.100, *p* < 0.001. Based on the tolerance and VIF values, no collinearity problems were observed.

## 4. Discussion

This study investigated the prevalence and the factors associated with social anxiety. Surprisingly, the prevalence of social anxiety was quite high (28.7%) compared to the study of social anxiety symptoms among participants aged 40–60 years old among the Omani population in 2022 (10.5%) [45]. Since the SIAS is a self-report questionnaire, the results should be considered indicative rather than definitive. A definite diagnosis of social anxiety disorder requires an interview with clinicians [46].

According to our findings, we found that social anxiety symptoms were associated with marital status, income, attachment anxiety, attachment avoidance, extraversion, perceived social stress, depression, and loneliness. Among all predictors, depression had the highest effect size (β = 0.242). 

Perceived social stress plays a vital role in social anxiety in this study, consistent with the findings of related studies, which indicated that perceived social stress in the teacher population is associated with factors such as workload, student behavior, employment, and financial status [47]. Lazarus and Folkman’s stress and coping model posits that stress was exacerbated in the face of challenging events when individuals felt overwhelmed and lacked the resources to cope, and research showed that perceived social stress was a positive predictor of anxiety and depression, suggesting that elevated levels of perceived social stress correspond with an increased likelihood of symptoms associated with anxiety and depression [48,49,50]. Combined with the existing literature, it appears that teacher stress is related to work and school [1], and financial stability [51]. These factors may interact to exacerbate teachers’ social anxiety levels. 

This current study found that depression and social anxiety symptoms were positively correlated and consistent with the findings of previous studies [26,52]. The prevalence of major depressive symptoms and anxiety symptoms was reported to be particularly high among teachers (16% and 43%) [1,53], and a study in Thailand in 2021 also found that 11.2% and 3.2% of teachers experienced severe or extremely severe anxiety and depression [54]. Teachers with depression were more likely to experience social anxiety across all domains, including social fears, avoidance behaviors, and physical symptoms [26], meaning that people with depression have psychological factors associated with social anxiety symptoms, such as negative thought patterns, self-denial, and social avoidance behaviors, which may lead to mutual reinforcement between the two. Secondly, during the middle-aged stage [55], individuals experience depression about aging and the future because of their experiences and age-related changes, such as physical health problems, feelings of loneliness, and shifting social roles, which leads to individuals’ limited perspective of future time, avoiding social interaction, and worrying about being unrecognized in a fast-paced society [56]. Notably, even though both anxiety and depressive symptoms were significantly related to social anxiety, anxiety symptoms became non-significant when controlling for other variables. 

As hypothesized, predisposing factors such as insecure attachment played a significant role in social anxiety, which was consistent with the findings of previous studies [15,16,17]. Adults with high levels of attachment anxiety had intrinsically negative perceptions and doubted their worthiness to receive support in everyday social and group interactions, questioned their ability to cope with distress, and showed fear of negative interpersonal outcomes [13], whereas people with high levels of attachment avoidance had high levels of self-criticism [57], showed distrust in others [58], and were uncomfortable with close relationships [59]. A high level of attachment anxiety suggests a preoccupied and fearful attachment, whereas a high level of attachment avoidance suggests a fearful and dismissing style [33]. In line with related studies, social anxiety is more strongly related to attachment anxiety than attachment avoidance [60,61]. This is because individuals with social anxiety desire social interaction but fear engaging in it.

While neuroticism is generally associated with stress and depression, extraversion was found to be strongly correlated with social anxiety symptoms, which was consistent with the findings of previous studies [20,62]. Extraversion describes active people who are sociable, talkative, and confident [63]. A 2009 study found that a teacher’s personality was correlated with his or her teaching anxiety and teaching strategies. Teachers with extroverted personalities were more likely to use student-to-student discussions and group work, and the teacher was also more pleasant to be around and had lower anxiety. This was because one of the main characteristics of extroverted personalities was the desire for social interaction, so extroverts would initiate classroom discussions and interactions and encourage the students to participate actively, thus negatively relating to social anxiety [64].

We found a positive correlation between loneliness and social anxiety symptoms, which was confirmed by previous reports [65,66]. If teachers experience loneliness, it may increase their risk for social anxiety, in turn exacerbating their feelings of social isolation [67]. Compared to young people, middle-aged people are at higher risk of social isolation and participate in fewer social activities, which makes them prone to loneliness, physical and cognitive decline, shifts in social roles, and poorer social adjustment. That is, negative social contact can cause middle-aged people to have a negative attitude and have feelings of loneliness, resulting in poor social adjustment and generating fewer social resources and support [68]. 

This study indicated that job burnout in middle-aged secondary schoolteachers was considered low. The reason might be that job burnout is reported as more common in younger staff [69], while all teachers in this study were in the middle age group. We did not find an association between job burnout and social anxiety symptoms. Although a previous study found that social anxiety was a significant predictor of job burnout [8], a bidirectional relationship was not confirmed. Although teachers in Thailand are required to do administrative work and are intensely evaluated on their teaching performances, the teachers in this study reported low levels of anxiety and moderate intensity of teaching evaluation. In contrast to the previous literature [21], we did not find any associations between neuroticism and social anxiety symptoms in these populations. One possible explanation might be that the effect of neuroticism on social anxiety symptoms was hindered by other factors included and not included in this study, such as comorbidities and positive mental health factors (resilience, perceived social support, and inner strength). The association between anxiety and social anxiety symptoms was not significant in the studied population.

### 4.1. Nonclinical Implications

According to the findings of this study, prevention and treatment should be provided to teachers suffering from social anxiety in the workplace by addressing depression and perceived stress as these conditions have a great impact on social anxiety symptoms. School management should pay attention to teachers’ work stress, reduce excessive workload, and provide teachers with a comfortable working environment and adequate resources to help them better cope with their work challenges [1]. In addition, schools could share self-efficacy skills and stress control among teachers through regular meetings and develop prevention/training programs to improve stress management skills [1]. Moreover, teachers’ collective efficacy as a work resource, and organizational policies that provide teachers with adequate social support may be an effective strategy for preventing and reducing teachers’ anxiety and depressive symptoms [70]. Finally, mental health counseling can be provided for those who need to better deal with stress and depression.

### 4.2. Clinical Implications

Our findings have important implications for guiding practice in related fields, particularly educational management, and mental health services. Those at risk of social anxiety and depression should be encouraged to receive evaluation from clinicians leading to early intervention. Additionally, a short questionnaire on attachment (10-item ECR-R) can be used as a screening tool to identify individuals at risk for developing stress, loneliness, social anxiety, and depression, enabling early intervention. Understanding and effective identification are crucial steps before implementing a strategic plan to reduce mental health issues and foster the well-being of middle-aged teachers.

## 5. Strengths and Limitations

This study has several strengths. First, it fills the knowledge gap in previous research on social anxiety symptoms and their associated factors among secondary schoolteachers, providing new perspectives and understanding in this area of research. Second, we synthesized multiple factors, such as personal characteristics, work-related factors, and psychological factors, which provide a more comprehensive explanation and understanding of social anxiety. 

Limitations of this study include sample limitations, data collection methods, study design, and unconsidered variables. First, our sample was drawn from secondary schoolteachers in Chiang Mai, Thailand, and therefore, might not be representative of teacher populations in other regions in Thailand or other counties, which limits the generalizability of the results. Second, we used a self-reported questionnaire to collect data, which may suffer from recall bias and self-report bias, resulting in compromised reliability and validity of the results. In addition, because this study used a cross-sectional design, causality could not be inferred, and we could not rule out the influence of potential confounders on the results. Finally, we did not consider potential influences such as avoidant personality traits or disorders, work environments, working duration, or personal health status, which may affect the interpretation and inference of the results. Future studies could use more samples, long-term follow-up designs, and control for more potential confounding variables to understand better the relationship between social anxiety symptoms and their associated factors. 

## 6. Future Directions

Based on the findings of this study, we proposed the following research directions to further explore and improve social anxiety symptoms in the teacher population. Firstly, future longitudinal studies could explore changes in social anxiety symptoms and their long-term effects among teachers at different career stages. In addition, a longitudinal study could help establish the causal relationship between the social anxiety symptom and other associated factors, especially burnout, depression, and perceived stress. Second, social anxiety symptoms among secondary schoolteachers across Thailand could be explored so that the data would be more generalized and representative. Thirdly, further research on environmental factors in the workplace could explore the effects of work hours and work experience, schoolwork environment, leadership support, collegiality, and student behaviors on teachers’ social anxiety symptoms, which could lead to specific strategies for improvement. Fourthly, the effectiveness of different psychological interventions in reducing teachers’ social anxiety symptoms could be assessed, such as which positive thinking training and social support groups are most effective in reducing teachers’ social anxiety symptoms.

## 7. Conclusions

A high proportion of middle-aged secondary schoolteachers exhibited social anxiety symptoms. Depression, loneliness, perceived social stress, and predisposing factors, i.e., extraversion, attachment anxiety, and attachment avoidance, were significant predictors of social anxiety symptoms. Early detection and appropriate interventions addressing the risk factors should be implemented to support the teachers in managing social anxiety symptoms. Further research using longitudinal studies is encouraged to document a causal relationship between social anxiety and those predictors.

## Figures and Tables

**Table 1:** Participants’ psychological measures (*n* = 341).

Variables	*n* (%)
Scores of Psychological Measures	Mean (SD ^1^)
Attachment	
- Attachment anxiety (range 5–35) - Attachment avoidance (range 5–35)	
Personality trait	
- Extraversion (range 8–32) - Neuroticism (range 8–32)	23.65 (4.25) 17.62 (5.75)
Anxiety and depression	
- Anxiety (range 0–24) - Depression (range 0–20)	7.99 (5.44) 3.54 (3.96)
Loneliness (range 6–24)	12.50 (4.88)
Perceived stress (range 0–40)	16.63 (8.57)
Job burnout (range 10–70) - Presence of job burnout (mean score >4)	25.47 (12.90) 43 (12.6)
Teaching evaluation	3.16 (1.72)
- Intensity of teaching evaluation (range 0–4) - Anxiety level of teaching evaluation (range 0–4)	2.01(1.04) 1.16 (1.04)
Social anxiety symptoms-Total (range 0–48)	8.92 (9.42)
- Social interaction anxiety scale (range 0–24) - Social phobia scale (range 0–24) - Social anxiety symptom (SIAS ≥ 7 and SPS ≥ 2), *n* (%)	4.51 (4.63) 4.41 (5.06) 98 (28.7)

^1^ Standard Deviation

**Table 2:** Social anxiety symptoms based on the participant’s characteristics (*n* = 341).

Demographic Characteristic	*n* (%)	SIAS and SPS Total Score	Test Difference ^6^	With Social Anxiety Symptoms *n* (%)	Without Social Anxiety Symptoms *n* (%)	Test Difference ^6^
		Mean ± SD ^1^	*p*-Value	*n* (%)	*n* (%)	*p*-Value
Age						
45–49	175 (51.3)	9.78 ± 9.19	0.187	57 (32.6)	118 (67.4)	0.176
50–54	86 (25.2)	8.41 ± 9.66		24 (27.9)	62 (72.1)	
55–59	80 (23.5)	7.58 ± 9.58		17 (21.25)	63 (78.75)	
Sex						
Male	106 (31.1)	10.38 ± 9.92	0.055	38 (35.8)	68 (64.2)	0.051
Female	235 (68.9)	8.26 ± 9.13		60 (25.5)	175 (74.5)	
Education						
Bachelor’s degree or lower ^2^	216 (63.3)	9.88 ± 9.66		72 (33.3)	144 (66.7)	
Master’s degree or higher ^3^	125 (36.7)	7.26 ± 8.79	0.011	26 (20.8)	99 (79.2)	0.014
Marital status						
Single ^4^	131 (38.4)	8.91 ± 9.24	0.988	40 (30.5)	91 (69.5)	0.563
In relationship ^5^	210 (61.6)	8.92 ± 9.55		58 (27.6)	152 (72.4)	
Income (Thai Baht)						
≤20,000	81 (23.8)	9.73 ± 9.44	0.376	26 (32.1)	55 (67.9)	0.444
>20,000	260 (76.2)	8.67 ± 9.42		72 (27.7)	188 (72.3)	

^1^: standard deviation. ^2^: secondary education (grade 9 or grade 12)/associate degree/vocational certificate/or equivalent/bachelor’s degree. ^3^: master’s degree/doctoral degree. ^4^: single/divorced/widowed/separated/break up. ^5.^ married and living together/married but not living together/not married but living together. ^6.^*t*-test or ANOVA.

**Table 3:** Correlation coefficients among mental health variables (*n* = 341).

Variables	1	2	3	4	5	6	7	8	9	10	11
1. Attachment anxiety	1										
2. Attachment avoidance	−0.103	1									
3. Neuroticism	0.405 **	−0.091	1								
4. Extraversion	−0.173 **	−0.232 **	−0.277 **	1							
5. Anxiety	0.425 **	0.009	0.638 **	−0.292 **	1						
6. Depression	0.386 **	0.068	0.555 **	−0.324 **	0.728 **	1					
7. Loneliness	0.381 **	−0.007	0.618 **	−0.329 **	0.643 **	0.640 **	1				
8. Perceived social stress	0.304 **	−0.122 *	0.522 **	−0.081	0.598 **	0.553 **	0.623 **	1			
9. Job burnout	0.391 **	−0.076	0.590 **	−0.257 **	0.739 **	0.740 **	0.659 **	0.599 **	1		
10. Teaching evaluation	0.163 **	−0.181 **	0.196 **	−0.004	0.291 **	0.227 **	0.105	0.127 *	0.223 **	1	
11. SIAS and SPS ^1^	0.356 **	0.192 **	0.446 **	−0.403 **	0.523 **	0.584 **	0.572 **	0.463 **	0.464 **	−0.075	1

^1^ SIAS and SPS, social interaction anxiety scale and social phobia scale, *: *p* < 0.05, **: *p* < 0.01.

**Table 4:** Multiple linear regression results of predictors of social anxiety symptoms.

Predictor	B ^1^	SE ^2^	β ^3^	*p*-Value	95% LL-CI ^4^	95% UL-CI ^5^	Collinearity Statistics Tolerance VIF	
(Constant)	−2.761	4.141		0.505	−10.909	5.386		
Age	−0.419	0.447	−0.038	0.350	−1.298	0.461	0.921	1.085
Sex	1.329	0.781	0.068	0.090	−0.209	2.867	0.934	1.071
Education	−0.926	0.803	−0.049	0.250	−2.505	0.654	0.831	1.203
Marital status	1.920	0.754	0.103	0.011	0.437	3.404	0.926	1.080
Income	1.904	0.943	0.087	0.044	0.049	3.758	0.813	1.230
Attachment anxiety	0.232	0.064	0.165	<0.001	0.106	0.359	0.731	1.369
Attachment avoidance	0.154	0.045	0.145	0.001	0.066	0.243	0.852	1.173
Extraversion	−0.385	0.095	−0.179	<0.001	−0.573	−0.198	0.771	1.298
Neuroticism	0.028	0.089	0.018	0.751	−0.147	0.204	0.480	2.084
Anxiety	0.017	0.115	0.010	0.885	−0.209	0.242	0.334	2.996
Depression	0.555	0.156	0.242	<0.001	0.248	0.862	0.327	3.056
Loneliness	0.340	0.122	0.182	0.006	0.099	0.580	0.355	2.814
Perceived social stress	0.252	0.061	0.235	<0.001	0.131	0.373	0.461	2.169
Job burnout	−0.052	0.052	−0.071	0.314	−0.154	0.050	0.307	3.261
* Adjusted R^2^ = 0.511, F (14, 309) = 25.100, *p* < 0.001								

^1^ Unstandardized coefficient, ^2^ standard error, ^3^ standardized coefficient, ^4^ lower limit confidence interval, ^5^ upper limit confidence interval, and * coefficient of determination (adjusted R square).

## Data Availability

The data presented in this study are available upon request from the corresponding author. Due to ethical restrictions, they are not publicly available.
